# The U-shaped crime recovery during COVID-19: evidence from national crime rates in Mexico

**DOI:** 10.1186/s40163-021-00147-8

**Published:** 2021-06-30

**Authors:** Jose Roberto Balmori de la Miyar, Lauren Hoehn-Velasco, Adan Silverio-Murillo

**Affiliations:** 1 Business and Economics School, Universidad Anahuac Mexico, Av. Universidad Anáhuac 46, 52786 Huixquilucan, Mexico; 2 Andrew Young School of Policy Studies, Georgia State University, Atlanta, USA; 3 School of Government, Tecnologico de Monterrey, Mexico City, Mexico

**Keywords:** Crime, Pandemic, Lockdown, COVID-19, Mexico

## Abstract

The existing empirical evidence suggests a reduction in aggregate crime as a consequence of the COVID-19 lockdown. However, what happens when lockdown measures are relaxed? This paper considers how the COVID-19 pandemic affects crime rates throughout Mexico when the stay-at-home orders end. We use national crime data from Mexico’s *National Public Security System*, which reports municipality-level rates on assault & battery, theft & property crime, fraud, drug crimes & extortion, and homicides. Our results show that the majority of crimes follow a U-shaped trend—when the lockdown ends—crimes rise back to pre-pandemic levels.

## Introduction

Pandemics fundamentally change the way human beings interact. The COVID-19 pandemic is no exception. For instance, adjustments in employment conditions have led to a significant share of individuals to transition to remote work. Likewise, individuals have transitioned their purchases to e-commerce transactions rather than brick- and-mortar stores. Worldwide crime patterns have also adjusted and show an evident decline during the COVID-19 pandemic (Ashby, 2020; Balmori de la Miyar et al., 2020; Hodgkinson & Andresen, 2020; Mohler et al., 2020; Poblete-Cazenave, 2020), especially through lockdowns that reshape criminal-victim interactions.

**Fig. 1:**
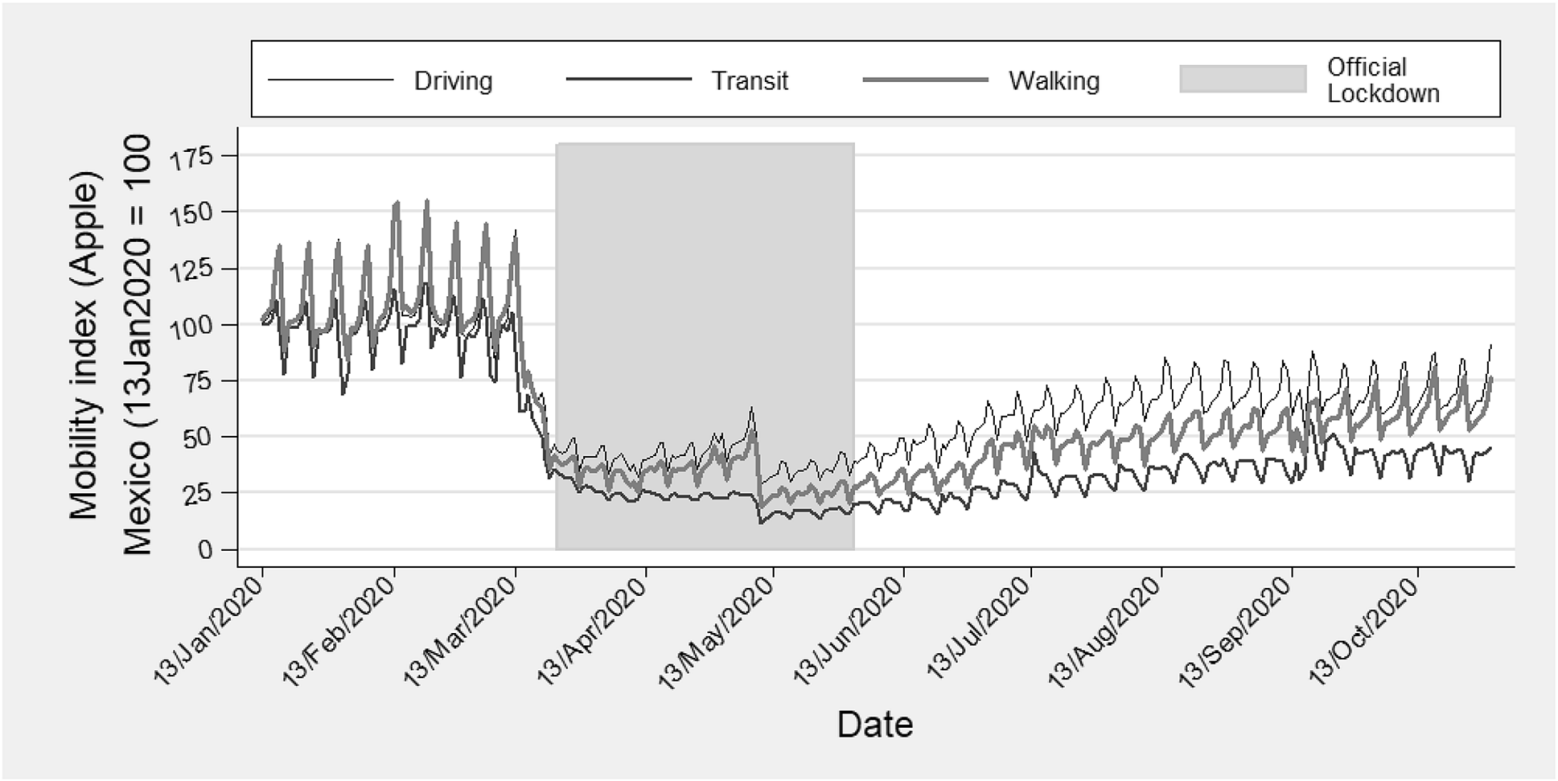
Mobility in Mexico. Source: APPLE MOBILITY INDEX

Mexico ranks as one of the countries experiencing the worst COVID-19 outbreaks (Johns Hopkins, 2021). Mexico’s initial cases appeared in early March 2020, and Mexico entered into a national lockdown from late March 2020 to May 2020. Beginning in June, restrictions eased to the local and state level. As shown in Fig. 1, mobility in Mexico declined after the beginning of the contagion stage, plummeting even more during the official national lockdown. Mobility recovered slightly after easing restrictions, through October 2020 (the conclusion of our analysis). Moreover, Mexico is a country with high criminal activity due to a decade-long drug war, leaving a severe toll in terms of human lives and violence. Under such circumstances, Mexico offers a unique context to explore whether non-structural events such as a pandemic alter criminal activity, permanently or temporarily.

In this study, we analyze the dynamic effect of the COVID-19 pandemic on crime rates in Mexico, during and after the national lockdown. We use municipality-level data from Mexico’s *National Public Security System* (NPSS). This national data includes a balanced panel of all Mexican municipalities for 2019 and 2020. The NPSS data contains crimes prosecuted by the States’ Attorneys General, including assault & battery, theft & property crime, fraud, drug crimes & extortion, and homicides. We group crimes by object: crime against property or crime against persons, and by victim-criminal proximity: strangers or acquaintances. In addition, we group all crimes to show the pandemic’s effect on crime (at large). To create crime rates, we combine the crime counts with population data from the *National Population Council*.

Employing a monthly series of crime rates, we apply an event-study design to describe how the pandemic affects crime and the trajectory of the effect during and after the national lockdown. We use the inter-temporal variation in criminal activity from January to October over two different years, 2019 and 2020. We use 2019 as a comparison for 2020, given that in 2019 there was no COVID-19 contagion in Mexico, and most federal and local authorities were the same as in 2020.

Our findings show two dominant patterns. First, assault & battery, theft & property crime, and fraud, decline and rise again after the national lockdown lifts. These crimes, as well as our aggregate measure of all crimes, follow a clear U-shaped pattern, with the lowest crime rates happening over the lockdown months of the pandemic (e.g., April and May). By October, seven months into the pandemic, most of the afore mentioned crimes are near or at pre-pandemic levels. Second, the more severe crimes, including drug crimes and extortions as well as homicides, all show minimal changes. Petty drug crimes and extortion slightly decline; however, these crimes return to pre-pandemic levels just after three months into the pandemic. All findings are robust to alternative specifications, including a bounding methodology and the exclusion of Mexico City, the largest urban area in Mexico.

Next, we turn to heterogeneity in the crime reduction. We split municipalities by whether the municipality had higher population and higher crime rates. Our results suggest that the most considerable crime reductions were in the most populous municipalities and in municipalities with high crime. Greater reductions in the most populous areas indicate that the lockdown may have reduced the likelihood of a victim-criminal match. We also consider differences by changes in state-level employment due to COVID-19. The results appear similar across both high and low unemployment states.

The remainder of this paper proceeds as follows. In "Theoretical reasons for the impact of COVID-19 on crime" section, we discuss the theoretical reasons for changes in crime. In "Related literature on the crime during the COVID-19 pandemic" section, we review the existing literature analyzing the relationship between COVID-19 and crime. Sections "Data" and "Empirical Strategy" describe the national crime data and empirical methods. Section "Results" presents the main findings, a series of robustness tests, and the heterogeneous effects. Section "Discussion" analyzes the implication of our results for policymakers. Section "Conclusion" concludes.

## Theoretical reasons for the impact of COVID-19 on crime

Restrictions on mobility resulting from the COVID-19 lockdown affects criminal activity (Gerell et al., 2020; Halford et al., 2020; Hodgkinson & Andresen, 2020). We consider four non-mutually exclusive theories for why this might be the case in Mexico. First, the reduction in economic and social activities outside of the household reduces the number of opportunities for victim-criminal interactions (Cohen & Felson, 1979). We know from mobility data that the COVID-19 pandemic led to a substantial decrease in economic and social activities outside of the household, as shown in Fig. 1. During the national lockdown, Mexico itself experienced a 70% mobility decline (Apple, 2020). The mobility decline may lead to fewer opportunities for victim-to-criminal interactions, resulting in lower crime rates (Cohen & Felson, 1979; Miller & Blumstein, 2020).

Second, in addition to the mobility reduction, criminals may be hesitant to engage in criminal activities due to fear of infection, even beyond restrictions imposed by strict lockdowns. Boman and Gallupe (2020) propose that the lockdowns specifically lower crimes that are committed in groups. Despite this reduction in group crimes, Boman and Gallupe (2020) suggest more severe crimes such as homicides fail to decline, and the reduction in previously petty crimes may be offset by the increased opportunity for intimate partner violence.

Third, criminal activity may decline due to an increase in criminal’s pro-social behavior. Fritz (1996) suggests that crime may fall after a catastrophic event due to the altruistic behavior of criminals. In this case, criminals engage in pro-social behavior to engender a "therapeutic community," promoting social cohesion across classes. If the therapeutic-community effect occurs during the pandemic, social cohesion, including pro-social behavior, should increase. This improved social cohesion may result in falling crime rates. Reicher and Stott (2020) argue that the sense of renewed community during the outbreak is one of the greatest resources for dealing with the crisis and can be used in policing strategies. Further, Jones (2020) also suggests that this time of crisis is an opportunity to build up the legitimacy of the police.

Fourth, the economic impacts of the COVID-19 pandemic may increase crime. Hoehn-Velasco et al. (2020) show that households in Mexico were severely affected by the COVID-19-related recession. The authors document that over the first three months of the pandemic, individuals in Mexico lost one-third of their income and nearly 20% of individuals lost employment (Hoehn-Velasco et al., 2020). The results in Hoehn-Velasco et al. (2020) apply to jobs in the formal and informal sectors, partly explaining the drop in mobility in Mexico. Despite this profound job loss, Hoehn-Velasco et al. (2020) find that Mexico’s government did not offer new public policies to aid affected groups. Moreover, Mexico has fewer remote work opportunities than high-income countries, weaker public support systems, and a larger informal sector. These poor economic conditions may increase criminals’ willingness to commit crimes for economic gain (Hagan, 1993).

Previous work demonstrates the link between unemployment and higher crime rates. In the United States, declines in unemployment result in reductions in crime (Raphael & Winter-Ebmer, 2001), with property crimes most affected. Further, local labor market options influence crime rates (Gould et al., 2002). These findings from Raphael and Winter-Ebmer (2001) and Gould et al. (2002) suggest that the lockdowns may increase crime through higher unemployment and reduced labor market opportunities. The literature also shows the importance of unemployment for within household crime. Bhalotra et al. (2019) find that an increase in men’s unemployment is associated with an increase in violence against women.

## Related literature on the crime during the COVID-19 pandemic

There is an emerging literature on the impact of the COVID-19 pandemic on crime throughout the world. In general, the evidence suggests a decrease in overall crime (Stickle and Felson, 2020), with heterogeneous effects by crime type. Namely, the literature shows the following three patterns of the COVID-19 lockdown on crime. First, the empirical evidence consistently suggests a decrease in property crimes, notably theft, robbery, and burglary. Second, there is mixed evidence for assault, battery, drug crimes, and homicides. Third, for the length of the crime drop, Andresen and Hodgkinson (2020) and Borrion et al. (2020) each find that crime returns back to normal levels after the initial decline.

**Evidence from the United States** In the United States, several studies across multiple cities find declines in robbery, theft, and burglary over the initial pandemic. Abrams (2021), using data for 25 large cities, demonstrates that crime drops over 35%, with the highest reductions related to crimes of theft and residential burglaries. Ashby (2020), employing data from 16 large cities, observes reductions in residential burglaries. Mohler et al. (2020), using data from Indianapolis and Los Angeles, discover a decrease in robbery and burglary.

Studies using single cities also find similar impacts on robbery, theft, and burglary. Campedelli et al. (2020a) see a decrease in robbery, shoplifting, and theft in Los Angeles. Shayegh and Malpede (2020) show a reduction in theft in Oakland and San Francisco. Felson et al. (2020) find a reduction in residential burglaries in Detroit. Campedelli et al. (2020b) find that roughly 13% of communities across Chicago experience reductions in burglaries and robberies.

Drug crimes in the United States also show a clear drop during the pandemic. Abrams (2021) finds a decline in drug crimes. Similarly, Campedelli et al. (2020b) show a decrease in narcotics-related offenses in Chicago in 45% of communities. These same studies in the United States find mixed effects on severe crimes, including homicides and assault and battery. Abrams (2021), Ashby (2020), Campedelli et al. (2020a) each find no decline in homicides, and Mohler et al. (2020) find no effect on assault-battery. By contrast, Shayegh and Malpede (2020) show a reduction in homicides after two weeks of the stay-at-home order, and Campedelli et al. (2020b), Halford et al. (2020), Campedelli et al. (2020a) show reductions in assaults. Overall, there is a less clear apparent relationship of the pandemic on violent crime.

**Evidence from Outside the United States** The same reductions in theft, burglary, and property crime appear outside the United States. In Europe and Canada Hodgkinson and Andresen (2020); Gerell et al. (2020); Halford et al. (2020) all find a reduction in theft. Hodgkinson and Andresen (2020), using crime data from Vancouver, present evidence of a decrease in theft. Gerell et al. (2020), employing data from Sweden, find a reduction in burglaries. Halford et al. (2020), using data from one U.K. police area, detect a decline in burglary and vehicle theft.

In India, China, Australia, and Mexico City, the reductions in theft, burglary, and robbery are similarly present. Poblete-Cazenave (2020) finds that the COVID-19 lockdown decreases burglary and robbery in Bihar, India. Borrion et al. (2020) demonstrate a reduction in theft in China. However, after the initial pandemic-related closures, Borrion et al. (2020) find that theft returned to higher than expected levels. In Australia, Andresen and Hodgkinson (2020) suggest a reduction in most types of crimes (excluding drug offenses). Finally, Balmori de la Miyar et al. (2020), using data from Mexico City, find a reduction in burglary and vehicle theft during the first weeks after the lockdown.

Unlike in the United States, where drug crimes decline, evidence from outside the United States does not suggest a reduction in drug crime. Gerell et al. (2020), using data from Sweden, suggest that narcotics-related crime is unchanged. Similarly, in Australia, Andresen and Hodgkinson (2020) find no change in drug-related offenses.

For more severe crimes, these findings also mimic the United States’ mixed findings. Calderon-Anyosa and Kaufman (2020) find a decrease in homicides in Peru. However, Balmori de la Miyar et al. (2020) demonstrate limited impact of the lockdowns on homicides in Mexico City. Balmori de la Miyar et al. (2020) do find a reduction in assaults.

## Data

We use municipal-level crime incidents throughout Mexico for 2019 and 2020 to measure the crime rate changes following the COVID-19 pandemic. The *National Public Security System* (*Secretariado Ejecutivo del Sistema Nacional de Seguridad Pública* or NPSS) collects the municipality-level crime data. The NPSS reports crime, including assault, battery, theft, property crime, fraud, petty drug crimes, extortion, and homicides.

We group crimes by object: crime against property or crime against persons, and by victim-criminal proximity: strangers or acquaintances. For instance, homicides are crimes against persons with close victim-criminal proximity. Assault & battery are also crimes against persons but without victim-criminal proximity. Petty drug crimes & extortions are property crimes with close victim-criminal proximity. While theft & property crime are property crimes without close victim-criminal proximity. Finally, fraud is a especially property crime where the victim may or may not know the offender. In addition, we group all crimes together to show overall effects.

The data is organized in a monthly series of municipalities. We consider the number of crimes per month per 100,000 inhabitants in each Mexican municipality. The number of municipalities in Mexico is 2457. The population data is added from the *National Population Council* (CONAPO), which reports municipality level population characteristics. The average population per municipality is 51,726 inhabitants. The smallest municipality has 87 inhabitants and the largest one has 1,859,266 inhabitants at the beginning of 2020. In the final sample, we include all Mexican municipalities over the course of the pandemic, from January to October, during both 2019 and 2020. We include 2019 as a comparison for 2020 to help capture seasonal trends. Thus, our final sample consists of 49,140 observations (2457 municipalities × 9 months × 2 years).

The timeline of COVID-19 related events in Mexico occurs in the following manner: the pandemic begins on March 11, 2020 as pronounced by World Health Organization (WHO, 2020). Mexico’s Council of General Health (CSG) announces the official stay-at-home order a few days after (March 23), even though mobility has already dropped substantially in Mexico one week before the official stay-at-home order (Apple, 2020; CSG, 2020). The official lockdown goes until May 30, 2020. Beginning in June, every state has to apply a traffic-light methodology, meant to ease the restrictions imposed during the confinement. Many businesses start to reopen, even though most schools and social clubs remain closed in Mexico. Figure 1 shows how mobility steadily moves back close to the pre-pandemic levels of the mobility index (13 January 2020 = 100).

## Empirical strategy

### Difference-in-differences specification

In our empirical strategy, we begin using a difference-in-differences specification to consider the effect of the COVID-19 pandemic on crime rates. This difference-in-differences strategy appears as:

1 $$Y_{mty} = \alpha + \beta \;{\text{Post-Lockdown}}_{mty} + \varphi_{m} t + a_{m} + \gamma_{t} + \nu_{y} + e_{mty}$$

where *Y*_*mty*_ is the outcome of interest for municipality *m* in month *t* and year *y*. Post-Lockdown_*mty*_ is a dummy variable that equals one for all the months in the post-lockdown phase in 2020. Post-Lockdown_*mty*_ is zero for the pre-lockdown months in 2020, and all of 2019. We also add *φ*_*m*_*t*, which are monthly municipality-level linear time trends. These trends account for linear growth in crime rates. *a*_*m*_ are municipality-fixed effects that control for time-invariant differences across municipalities. *γ*_*t*_ are monthly-specific fixed-effects. *ν*_*y*_ are year fixed effects. We cluster standard errors at the municipality level. To account for differences in the variance of the crime rate between high population and low population municipalities, we weight the specification by the population. The population weights account for small municipalities having potentially large fluctuations in the crime rate, while larger cities should have more stable crime rates.

### Event-study

We next study the dynamic time-varying effect of the COVID-19 pandemic on crime outcomes using a monthly event-study specification. Goodman-Bacon and Marcus (2020) propose that researches should also present event-study estimates when trying to analyze the causal effects of the COVID-19. In particular, the event-study has two advantages over the difference-in-differences methodology. First, the difference-in-differences strategy quantifies the *average* effect of the COVID-19 lockdown over the entire post-period. Yet, the event-study has the advantage to quantify the evolution of the lockdown month-to-month (Goodman-Bacon, 2018; Wolfers, 2006). Second, the event-study can help to test the "parallel trends" assumption which is necessary to validate the results of the difference-in-differences model. In particular, this assumption states that there are not pre-pandemic changes in the outcomes of interest.

To estimate the effect of the COVID-19 pandemic on crime, we use a monthly event-study specification. Our preferred specification is represented as follows:

2 $$Y_{mty} = \alpha + \sum\limits_{\begin{subarray}{l} q = - {2} \\ q \ne - {1} \end{subarray} }^{7} {\beta_{q} Lockdown_{mqy} + a_{m} + \gamma_{t} + \nu_{y} + e_{mty} }$$

where *Y*_*mty*_ is the outcome of interest for municipality *m*, for month *t*, and year *y*. *Lockdown*_*mqy*_ is a dummy variable that takes the value of one for each month *q* before and after the start of the lockdown for municipality *m* in 2020. In particular, the lockdown begins in March of 2020 (month three), but March is represented by *q* = 0 in the specification above. *q* =  *− *2 corresponds to two months before the lockdown or January of 2020. *q* =  *− *1 represents one month before the lockdown or February of 2020. Our specification continues until *q* = 7 which represents seven months after the lockdown, or October, 2020. Thus, *Lockdown*_*mqy*_ is a dummy variable where *q* ranges from-2 through 7.

Then, we follow the literature analyzing the effects of the COVID-19 using event studies (Bullinger et al., 2020; Hoehn-Velasco et al., 2021; Leslie & Wilson, 2020) and exclude the month before the pandemic occurred (*q* =  *− *1). This fulfills two objectives. First, it is necessary to exclude one period to avoid multicollinearity. In this literature, the period that is mainly excluded is that before the occurrence of the event. Second, it is necessary to include in the regression the information regarding the municipalities not impacted by the COVID-19 lockdown in 2019 (our control). We follow again the literature computing these municipalities with the value of *q* =  *− *1. When we exclude *q* =  *− *1, these values are captured in the coefficient associated to the constant in the regression *α*. Thus, the changes observed in the event-study are compared to the information regarding 2019.

Finally, *a*_*m*_ are municipality-fixed effects which control for time-invariant differences across municipalities; *γ*_*t*_ are monthly fixed-effects; and *ν*_*y*_ are year fixed effects. We cluster standard errors at the municipality level. The specification is weighted by the municipal-level population. Our primary coefficients of interest are the *β*_*q*_, which reflects the impact of the COVID-19 pandemic on crime rates over the course of the pandemic.

## Results

### Descriptive results

Table 1 shows the means of the crime rates over the pre-lockdown period (2019 and January and February of 2020) relative to the post-lockdown period (months March through October of 2020). The measures of all crimes fall substantially during the post-COVID-19 lockdown period. Total crime rates fall from 94 crimes per 100,000 inhabitants to 76 crimes per 100,000 inhabitants, reflecting a 20% reduction in crime.

**Table 1:** Descriptive statistics

	Pre-lockdown		Post-lockdown		Total	
	Mean	Std. Dev	Mean	Std. Dev	Mean	Std. Dev
All crime	94.076	66.683	76.767	52.822	87.120	62.072
Assault and battery	21.173	14.678	18.528	12.973	20.110	14.078
Theft and property crime	59.528	47.706	45.348	35.361	53.830	43.727
Fraud	5.062	6.939	4.587	6.147	4.871	6.636
Petty drug crime and extortion	5.391	9.135	5.461	10.280	5.419	9.611
Homicides	2.921	3.346	2.843	3.432	2.890	3.381
N	29,484		19,656		49,140	

Source: *Mexico’s National Public Security System* (*Secretariado Ejecutivo del Sistema Nacional de Seguridad Pública*)

Crime rates are measured per 100,000 inhabitants. The specification is weighted by the municipal-level population

For the specific crimes, the individual crimes’ means demonstrate that the lockdown has heterogeneous impacts across crime types. The majority of the reduction in crime arises from lower theft & property crimes. Theft & property crime falls from 60 incidents per 100,000 inhabitants to 45 per 100,000, a 25% drop in theft and property crime. Assault & battery exhibit the next largest drop in crime. Assault & battery falls from 21 crimes per 100,000 inhabitants to 18 crimes per 100,000, a 14% drop.

The remainder of the crime rates exhibit more minor changes. Fraud falls from 5 incidents per 100,000 to 4.5 per 100,000, a 10% drop. Drug crimes & extortion, by contrast, slightly increase after the lockdown from 5.4 to 5.5 per 100,000 inhabitants. Finally, homicides fall from 2.9 crimes per 100,000 inhabitants to 2.8 crimes per 100,000 persons, a 3% reduction.

### Difference-in-difference results

We begin the main results by showing our difference-in-differences estimates in Table 2. The difference-in-differences approach compares the post-period months’ March through October to the pre-pandemic months and all of 2019. The results suggest that all crimes decline by 17 crimes per 100,000 inhabitants. Homicides are the only type of crime that fails to decline in the post-pandemic period. Measures of other types of crime, including assault & battery, theft & property crime, fraud, as well as petty drug crime & extortion, all substantially decline.

**Table 2:** Difference-in-differences specification

	All crime	Assault & battery	Theft & property crime	Fraud	Drug crime & extortion	Homicides
	(1)	(2)	(3)	(4)	(5)	(6)
Post x Lockdown	− 16.979***	− 5.132***	− 9.985***	− 1.019***	− 0.836***	− 0.007
	(1.191)	(0.350)	(0.867)	(0.206)	(0.294)	(0.070)
Observations	49,140	49,140	49,140	49,140	49,140	49,140
Adjusted R-sq	0.93	0.77	0.93	0.79	0.89	0.49
Mean dependent	87.12	20.11	53.83	4.87	5.42	2.89
Baseline FE	X	X	X	X	X	X
Time Trends	X	X	X	X	X	X

Source: *Mexico’s National Public Security System* (*Secretariado Ejecutivo del Sistema Nacional de Seguridad Pública*)

Difference-in-differences estimates, which group the pre-period (before month 3) and post periods (month three onward). We include time trends in the difference-in-differences specification. Baseline fixed effects are included at the municipality, month, and year. Vertical lines show the start and end of the lockdown. The long-dashed red line shows the month before the start of the lockdown and the short-dashed green line indicates the end of the lockdown. Robust standard errors are clustered at the municipal level. Significance levels: *p < 0.1, **p < 0.05, ***p < 0.01 Crimes are measured per 100,000 persons. The specification is weighted by the municipal-level population

In particular, the COVID-19 pandemic reduces assault & battery rates by 5.1 crimes per 100,000 inhabitants. Similarly, theft & property crimes fall by ten crimes per 100,000 inhabitants. Fraud drops by one crime per 100,000 inhabitants. Further, petty drug crime & extortion decline by 0.8 crimes per 100,000 inhabitants. All of these coefficients are statistically significant at the 99% confidence level. Moreover, the adjusted R-squared statistic suggests our model in Eq. 1 describes most of the variation in crime rates.

### Event-study results

**Fig. 2:**
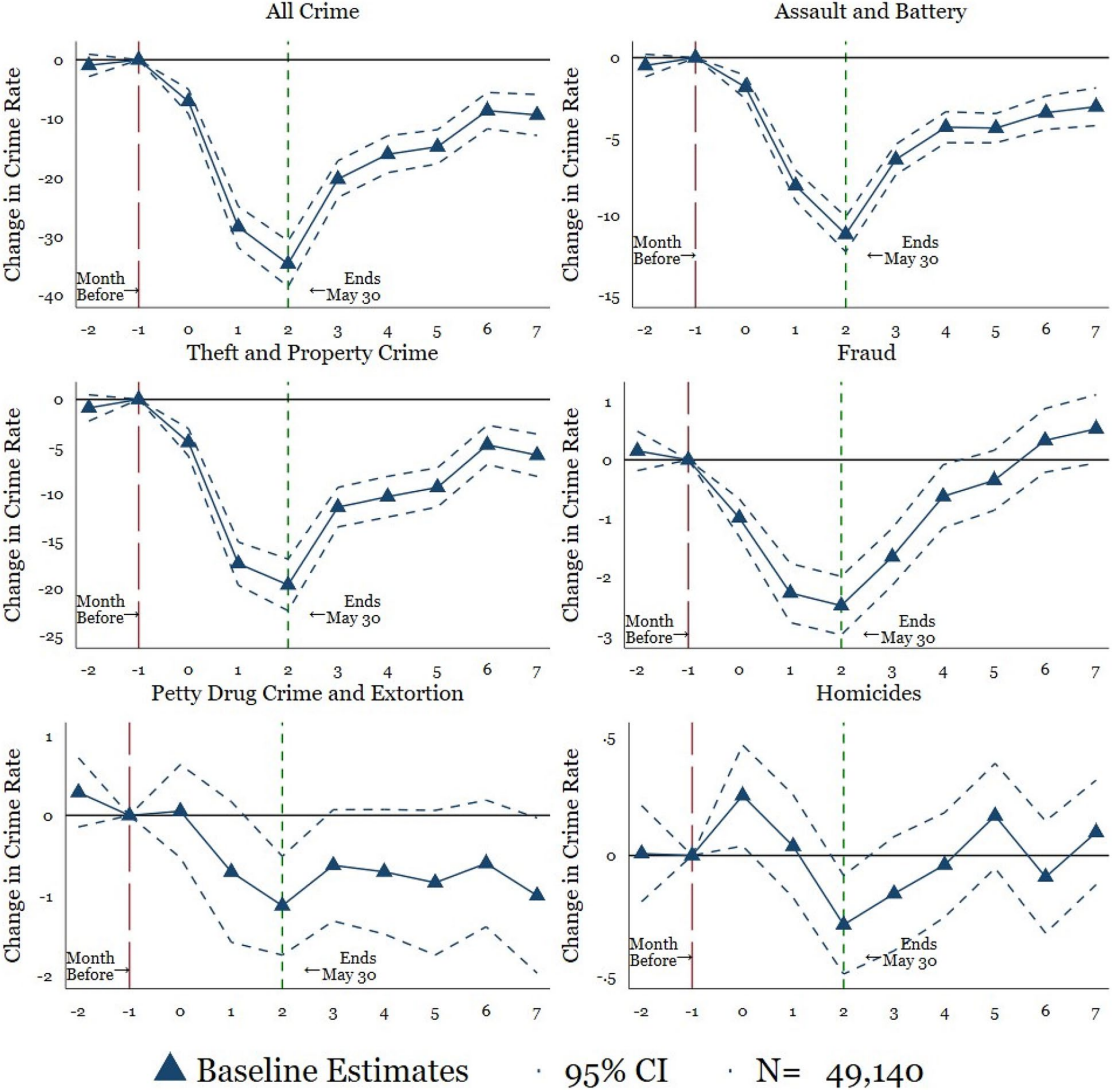
Event Study: Main Findings. *Mexico’s National Public Security System* (*Secretariado Ejecutivo del Sistema Nacional de Seguridad Pública*). Plotted coefficients are event-study dummy variables, *β*_*q*_. Each plotted point represents the number of months before and after the lockdown. Solid lines represent point estimates. Dashed and dotted lines display the 95 percent confidence intervals. Crimes are measured per 100,000 persons. Baseline fixed effects are included at the municipality, month, and year. Vertical lines show the start and end of the lockdown. The long-dashed red line shows the month before the start of the lockdown and the short-dashed green line indicates the end of the lockdown. The specification is weighted by the municipal-level population. Robust standard errors are clustered at the municipal level. See Table 4 for exact coefficients and standard errors

Next, Fig. 2 and Table 4 contain the results for Eq. 2, for our six primary measures of crime. The plotted points connected by solid lines show change in crime rates. In particular, the plotted points depict the point estimates of the difference between monthly crimes per 100,000 inhabitants in 2020 in comparison to the mean of the months not impacted by the COVID-19 pandemic in 2019 after controlling for municipality, monthly, and year fixed effects (see "Event-study" section). The dashed and dotted lines indicate the 95% confidence intervals around the point estimates. The long-dashed vertical red line indicates the excluded pre-period, which includes 2019. The short-dashed vertical green line, which in turn, represents the end of lockdown, and the recovery of mobility in Mexico (as shown in 1).

In the first graph of Fig. 2, we show the results for all crime rates. In the first few months of the lockdown, crime declines by 20 to 30 incidents per 100,000 inhabitants. When the lockdown ends on May 30th, crime rates start to rebound, nearly returning to normal by the end of the monthly series (October).

For the specific types of crime in the remainder of the graphs, there are heterogeneous effects across crime measures. On the one hand, assault & battery decline substantially after the lockdown. As the lockdown lifts in June, these two crimes begin to rise back to their original levels. However, assault & battery are relatively flat after four months into the pandemic (e.g., July, August, September, and October), and it never attains the pre-pandemic levels. Theft & property crime also drops quickly after the pandemic. Theft & property crime then begin to rise again but are relatively steady over months three to five into the pandemic (e.g., June through August), with another bump in month six (e.g., September). Fraud drops rapidly but makes the quickest recovery. Fraud is nearly back to pre-pandemic levels by month five of the pandemic (e.g., August), surpassing the pre-pandemic level in the last two months of the series.

On the other hand, petty drug crimes & extortion, in the fifth graph, follow a different pattern than the previous crimes and have a less noticeable dip and immediate rise back in months two and three into the pandemic. Next, we analyze the evolution of homicides. In the sixth graph, homicides fluctuates slightly during the first three months of the pandemic and then hovers around zero, suggesting an overall null effect. In Mexico, homicides as well as petty drug crimes & extortion are related to organized crime, and they do not appear to decline drastically during the pandemic.^1^ However, our data does not include information about whether these last crimes have a solo or co-offending modus operandi.

### Robustness checks

To test the robustness of our main event-study findings, we perform two additional checks on the main analysis. First, we show the sensitivity of our results using the Oster bounding methodology. Second, we explore the results, excluding Mexico City, which may differ from the rest of Mexico. These tests confirm the central theme of the results and boost confidence in the robustness of our findings.

First, we conduct the bounding methodology proposed by Oster (2017). Oster (2017)’s bounding method is a refined approach to the original bounding demonstrated in Altonji et al. (2005). This check assumes that the selection on observables is informative about the degree of selection on unobservables. Oster (2017) formalizes the bounding approach by providing conditions for bounds and identification. The exact approach sets a minimum for the R-squared from simulated regression with unobservables. If the simulated bounds exclude zero, then the results from the regression are robust to omitted variable biases.^2^ The results for the bounding approach are shown in Appendix Table 3, with the intervals in square brackets as the bounds. The bounds on the coefficients confirm the findings from the main results.

As a second robustness check, we exclude Mexico City. Mexico City is distinct in setting from the remainder of Mexico. Mexico City has higher crime levels and differing economic policies from Mexico at large. Figure 5 shows the results without Mexico City in navy. The point estimates without Mexico city are similar to the main findings. The reduction in crime does not appear to be isolated in Mexico City.

### Heterogeneous effects

Next, we test whether there are heterogeneous effects by municipality-level size or in municipalities with higher men’s employment losses. Thus, we split our sample between large/smaller municipalities. We also split our sample between higher/lower men’s employment losses. Then, we estimate the event-study (Eq. 2) for each sample.

**Fig. 3:**
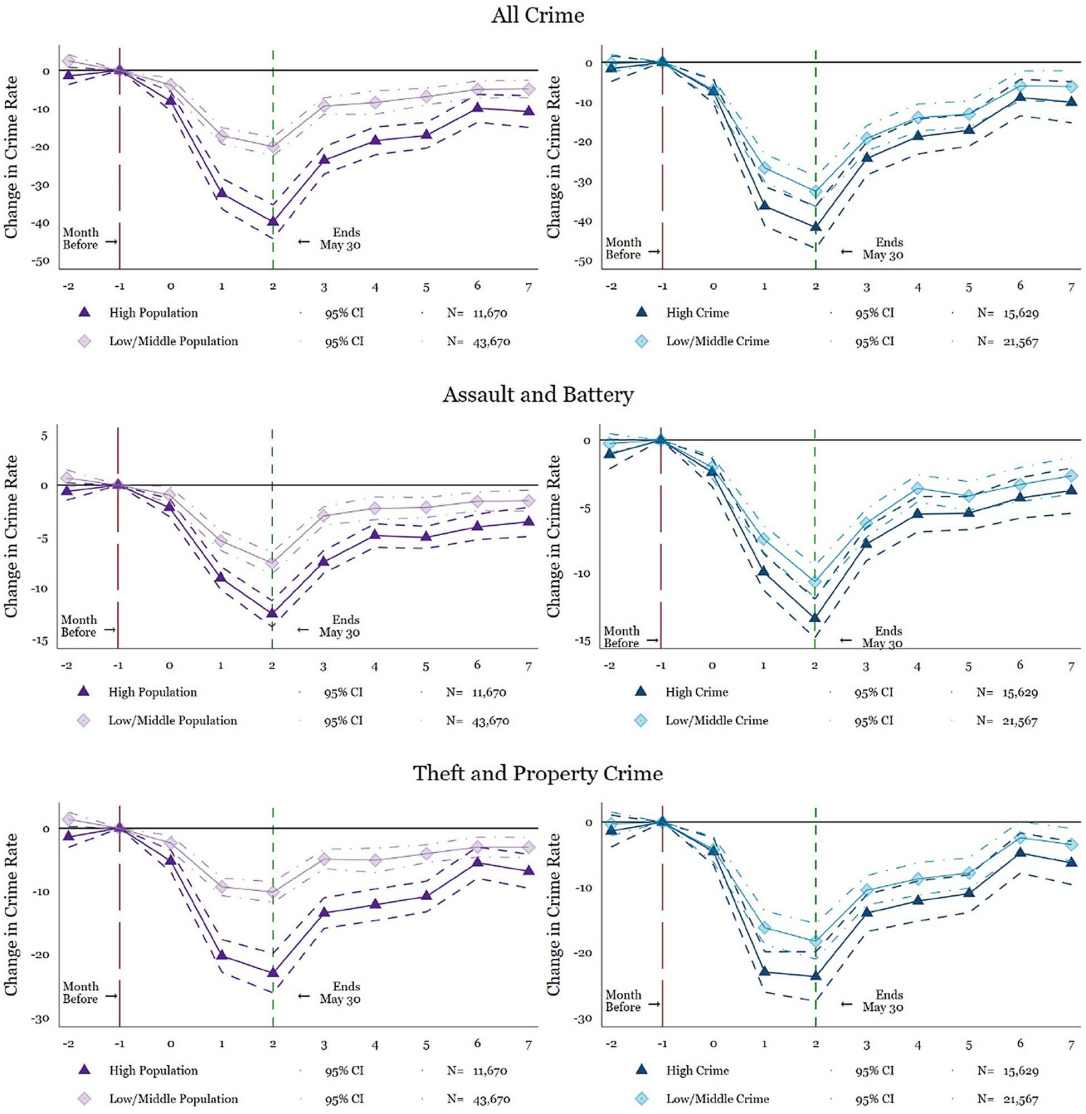

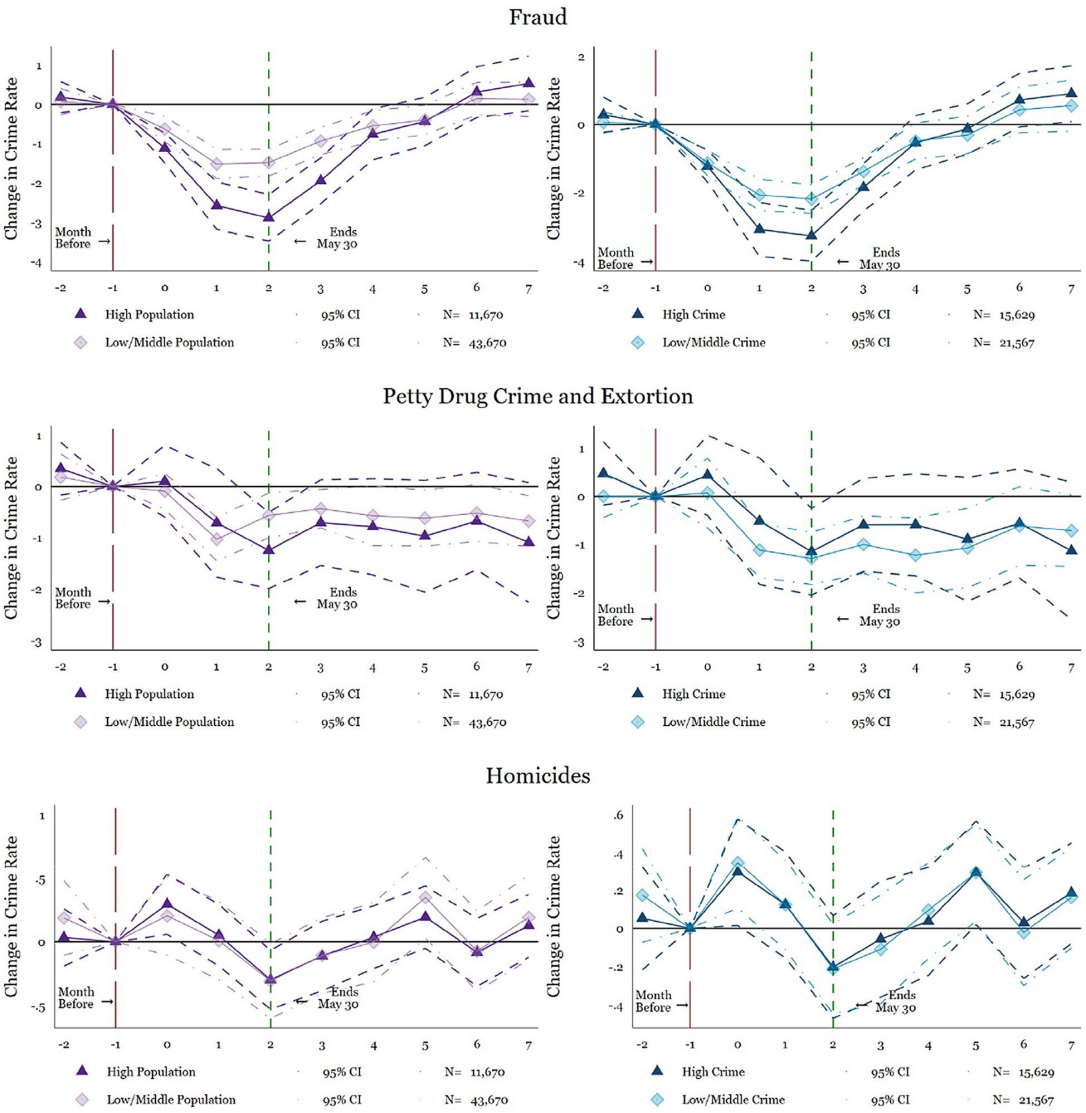
Event study: heterogeneity by population and crime levels. Source: *Mexico’s National Public Security System* (*Secretariado Ejecutivo del Sistema Nacional de Seguridad Pública*). Plotted coefficients are event-study dummy variables, *β*_*q*_. Each plotted point represents the number of months before and after the lockdown. Solid lines represent point estimates. Dashed and dotted lines display the 95 percent confidence intervals. Crimes are measured per 100,000 persons. Baseline fixed effects are included at the municipality, month, and year. Vertical lines show the start and end of the lockdown. The long-dashed red line shows the month before the start of the lockdown and the short-dashed green line indicates the end of the lockdown. The specification is weighted by the municipal-level population. Robust standard errors are clustered at the municipal level. Dark purple graphs in the first column shows high population municipalities. Light purple in the first column shows low/middle population municipalities. In the second column, dark blue shows high crime municipalities and light blue plots low/middle crime municipalities. See Tables 5, 6, 7 and 8 for exact coefficients and standard errors

First, we show the main event-study excluding the population and crime rate tails (top 5% and bottom 5%), as well as considering the municipalities with the highest crime rate and largest population sizes (top 25%). Figure 3 shows similar results to the main findings, with a few exceptions (see Tables 5, 6, 7, 8, for coefficient and standard error estimations). Excluding the highest population areas and focusing on the middle and low population areas, the plotted points suggest a smaller reduction in crime. The highest reductions in crime occur in the populous municipalities with the highest preexisting crime rates. These are exactly the municipalities where more victims would coincide with criminals under normal circumstances. This more evident drop in crime levels for the most populous cities supports the hypothesis of a reduction in the interaction between potential victims and offenders (Cohen & Felson, 1979).

**Fig. 4:**
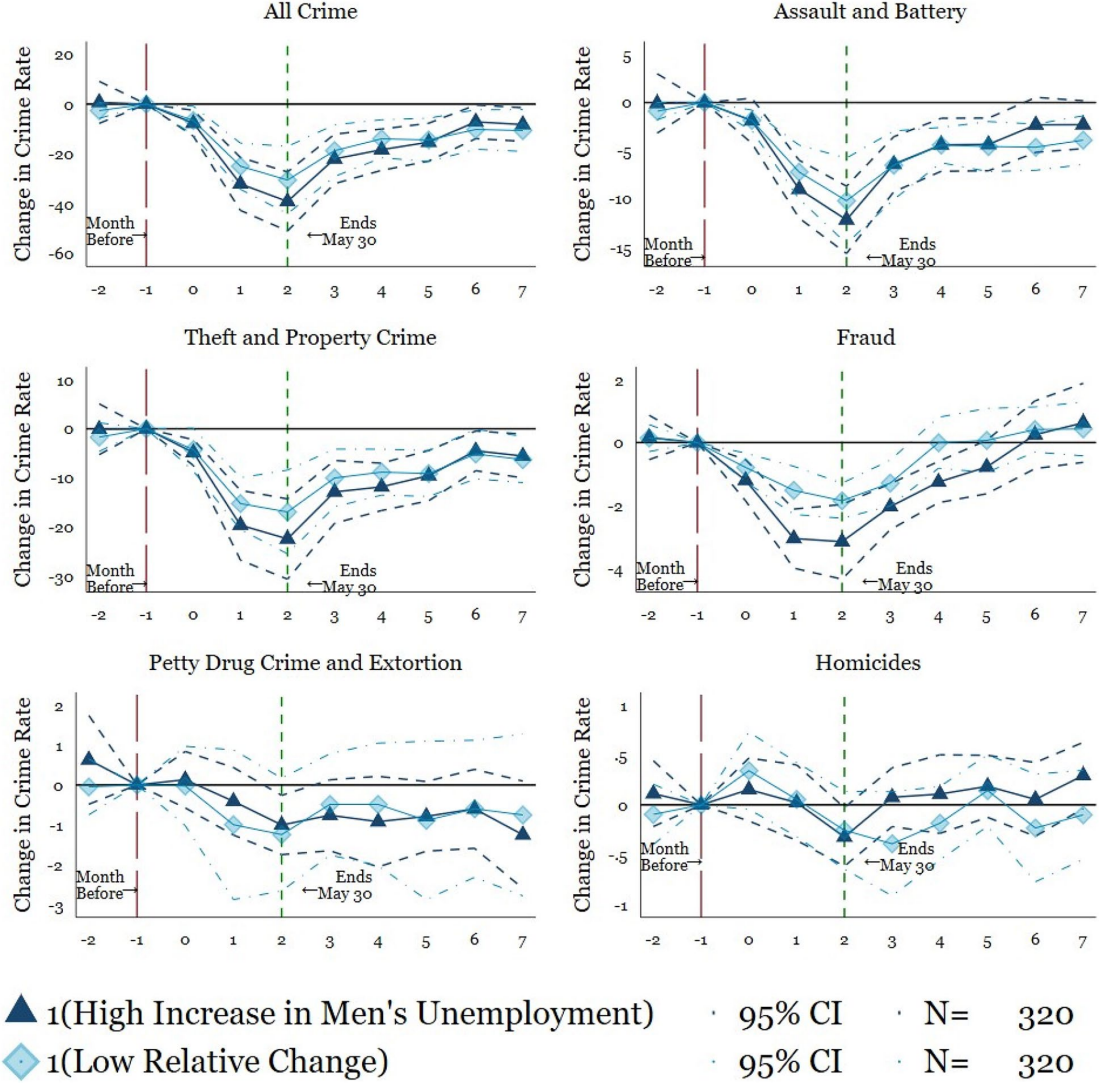
Heterogeneity by high and low relative change in men’s state-level unemployment. Source: *Mexico’s National Public Security System* (*Secretariado Ejecutivo del Sistema Nacional de Seguridad Pública*). Plotted coefficients are event-study dummy variables, *β*_*q*_. Each plotted point represents the number of months before and after the lockdown. Solid lines represent point estimates. Dashed and dotted lines display the 95 percent confidence intervals. Crimes are measured per 100,000 persons. Crimes are measured per 100,000 persons. Baseline fixed effects are included at the state, month, and year. Vertical lines show the start and end of the lockdown. The long-dashed red line shows the month before the start of the lockdown and the short-dashed green line indicates the end of the lockdown. The specification is weighted by the state-level population. Robust standard errors are clustered at the state level. See Table 9 and 10 for exact coefficients and standard errors

**Fig. 5:**
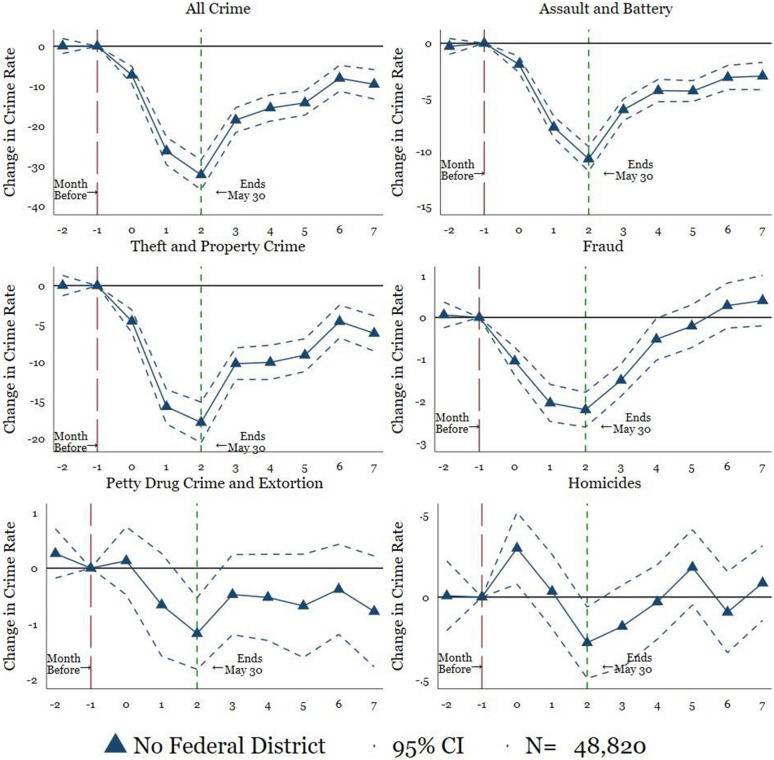
Event study: no Mexico City. Source: *Mexico’s National Public Security System* (*Secretariado Ejecutivo del Sistema Nacional de Seguridad Pública*). Plotted coefficients are event-study dummy variables, *β*_*q*_. Each plotted point represents the number of months before and after the lockdown. Solid lines represent point estimates. Dashed and dotted lines display the 95 percent confidence intervals. Crimes are measured per 100,000 persons. Baseline fixed effects are included at the municipality, month, and year. Vertical lines show the start and end of the lockdown. The long-dashed red line shows the month before the start of the lockdown and the short-dashed green line indicates the end of the lockdown. Robust standard errors are clustered at the municipal level. See Table 11 for table presenting the exact coefficients and standard errors

Second, based on the prior theoretical and empirical work linking unemployment to crime (Bhalotra et al., 2019; Gould et al., 2002; Raphael & Winter-Ebmer, 2001), we test whether higher unemployment affects the crime reduction. Figure 4 shows the states split into high and low unemployment rates (due to COVID-19 changes in employment) for men (see Tables 9, and 10 for coefficient and standard error estimations). The results appear similar across both high and low unemployment states. The only exceptions are fraud and theft & property crime which shows a steeper decline in high unemployment cities. Nevertheless, the confidence intervals are so wide that we cannot affirm a statistically significant difference between high and low unemployment states. Overall, the similarity across unemployment levels suggests that unemployment (at least at the beginning of the pandemic) does not play a large role in the crime rates. However, the difference across unemployment rates may change as individuals continue without financial support during the pandemic.

## Discussion

The decrease in crimes such as assault, battery, theft, property crime, and fraud, supports the victim-criminal interaction hypothesis (Cohen & Felson, 1979). Once the lockdown is in place, a sharp decrease in these crimes occurs (red vertical line). Nonetheless, when the confinement ends (green vertical line), many people returned to their normal activities, increasing the likelihood of a victim-crime interaction.

The Mexican government, as stated by the Minister of Security, claims that its current security strategy is successful because of an unprecedented decline in crime (Forbes, 2020). The current strategy (2019–2020) consists of easing down on the killings of cartel members ("hugs, not bullets"), and on putting more forces into the streets to discourage criminal activities, despite most of these forces being military personnel. And even though some investments are being made in terms of incorporating more forces into the streets of Mexico (Forbes, 2020), our results show that the decline in crime appears to be related to the pandemic lockdown.

Worse yet, in spite of the arrests of important leaders of cartels such as Joaquin "El Chapo" Guzman or José Antonio "El Marro" Yépez Ortiz, homicides, petty drug crimes and extortion do not show signs of dropping during the pandemic. This reflects the failure in the security strategy to fight drug cartels in Mexico. Moreover, the ongoing investigation of a former Mexican Defense Minister under President Peña, for its allegedly link with the Beltrán-Leyva criminal organization (Romo, 2020), or the arrest of the former Citizen Security Minister in Mexico under President Calderon, the architect of Mexican Drug War, for allegedly helping "El Chapo" and the Sinaloa Cartel (Feuer, 2020), exposes the structural weaknesses of the past and recent military-driven security strategies in Mexico.

Future research should try to distinguish whether the null effect of the COVID-19 pandemic on homicides, drug crimes, and extortion has to do with a difference between solo- and co-offending. Namely, crimes related to organized crime, such as homicides, drug crimes, and extortion, might be unchanged as the monetary incentives to participate in organized crime might be exceeding the risk of being infected by the COVID-19 virus. A limitation of our study is that we cannot make any claim about solo- or co-offending heterogeneity with our current data. However, fatality data published by Mexico’s Health Ministry with a two-year lag has the potential and the information to distinguish between solo- or co-offending homicides. Drug crime and extortion can also be analyzed using victimization surveys with enough information about the characteristics of the crimes during the pandemic period.

## Conclusion

In this paper, we study how crime rates throughout Mexico change during the COVID-19 pandemic. This public health crisis has the potential to cause criminals and victims to stay home, resulting in a reduction in victim-criminal interaction. We observe this hypothesis to be true for conventional crimes such as fraud, assault & battery, as well as theft & property crime. These crimes follow a U-shaped pattern, where crime falls precipitously and then rises back near the original crime levels after mobility recovers. By October, seven months into the pandemic, fraud has surpassed its pre-pandemic level, while assault & battery and theft & property crime are near their baseline levels. While the full 2020 effects are still unknown, we expect that the rise in conventional crime will continue, until most crimes reach or surpass original pre-pandemic levels.

Crimes rates from organized crime such as homicides, drug crimes, and extortions do not substantially change during the pandemic. Based on Boman and Gallupe (2020), we expected a reduction in organized crime because of the risk of infection when interacting in groups. Yet, it is possible that the monetary compensation provided by drug cartels over compensate the risk of infection. This is an important result for future research, when assessing how criminals weight risky decisions.

Last, we test several heterogeneous effects. First, we check whether the highest reductions in crime occur in the most populous municipalities and areas with the highest preexisting crime rates. Our results indicate that crime did decline by more in the most populous municipalities, supporting the victim-criminal interaction hypothesis. Second, we test whether unemployment affects the crime rate. We do not observe a clear difference in reductions in crime by the unemployment rate. Despite this lack of effect, in the long-term, unemployment could be a potential driver of rising crime in Mexico.

## Data Availability

All data are open source and available from the authors upon request.

## Appendix 1

See Table

**Table 3:** Oster’s bounding methodology

	All crime	Assault & battery	Theft & property crime	Fraud	Drug crime & extortion	Homicides
	(1)	(2)	(3)	(4)	(5)	(6)
Month − 2	[− 27.80, 1.32]	[− 0.89, 3.93]	[− 3.44, 0.46]	[− 0.01, 1.49]	[− 0.32, 0.49]	[− 0.05, 0.65]
Month 0	[− 67.72, − 2.48]	[0.44, − 25.19]	[− 6.48, − 3.50]	[− 0.66, − 3.70]	[− 0.54, 0.25]	[0.14, 0.25]
Month 1	[− 60.40, − 26.62]	[− 27.13, − 6.36]	[− 17.74, − 17.22]	[− 2.94, − 2.19]	[− 0.83, − 0.65]	[− 0.03, 0.86]
Month 2	[− 108.06, − 28.19]	[− 43.16, − 7.85]	[− 21.58, − 18.63]	[− 4.65, − 2.24]	[− 1.97, − 0.82]	[− 0.85, − 0.23]
Month 3	[− 56.16, − 17.67]	[− 30.26, − 4.09]	[− 11.45, − 11.37]	[− 5.62, − 1.17]	[− 1.30, − 0.38]	[− 0.17, 0.05]
Month 4	[− 72.88, − 12.74]	[− 23.92, − 2.52]	[− 11.84, − 9.49]	[− 4.74, − 0.12]	[− 2.09, − 0.21]	[− 0.06, 0.22]
Month 5	[− 76.49, − 11.16]	[− 26.63, − 2.27]	[− 10.39, − 8.75]	[− 4.56, 0.16]	[− 2.42, − 0.28]	[0.11, 0.79]
Month 6	[− 26.92, − 7.16]	[− 24.80, − 1.40]	[− 5.42, − 3.67]	[− 1.95, 0.60]	[− 1.44, − 0.30]	[− 0.15, 0.64]
Month 7	[− 71.01, − 4.12]	[− 28.25, − 0.56]	[− 7.33, − 5.14]	[− 4.71, 1.17]	[− 2.41, − 0.50]	[0.04, 0.73]
Baseline FE	Yes	Yes	Yes	Yes	Yes	Yes
Observations	49,140	49,140	49,140	49,140	49,140	49,140
*R*^2^	0.31	0.77	0.90	0.53	0.86	0.48

Intervals in squares are the bounds. Baseline fixed effects are included at the municipality, month, and year

3 and Fig. 5.

## Appendix 2: Event study tables for main figures

See Tables

**Table 4:** Event-study specification (Fig. 2)

	All crime	Assault & battery	Theft & property crime	Fraud	Drug crime & extortion	Homicides
	(1)	(2)	(3)	(4)	(5)	(6)
Month − 2	− 0.947	− 0.496	− 0.900	0.154	0.288	0.008
	(0.972)	(0.365)	(0.711)	(0.172)	(0.220)	(0.102)
Month 0	− 7.105***	− 1.866***	− 4.550***	− 0.993***	0.053	0.249**
	(1.075)	(0.380)	(0.742)	(0.166)	(0.296)	(0.107)
Month 1	− 28.449***	− 8.097***	− 17.408***	− 2.278***	− 0.706	0.039
	(1.786)	(0.494)	(1.168)	(0.258)	(0.447)	(0.109)
Month 2	− 34.748***	− 11.177***	− 19.663***	− 2.494***	− 1.126***	− 0.288***
	(1.989)	(0.563)	(1.397)	(0.255)	(0.314)	(0.105)
Month 3	− 20.317***	− 6.450***	− 11.426***	− 1.659***	− 0.623*	− 0.159
	(1.598)	(0.492)	(1.069)	(0.249)	(0.354)	(0.121)
Month 4	− 16.083***	− 4.400***	− 10.315***	− 0.625**	− 0.703*	− 0.039
	(1.602)	(0.499)	(1.102)	(0.276)	(0.396)	(0.111)
Month 5	− 14.809***	− 4.459***	− 9.326***	− 0.351	− 0.839*	0.165
	(1.484)	(0.472)	(1.059)	(0.261)	(0.460)	(0.111)
Month 6	− 8.652***	− 3.483***	− 4.814***	0.333	− 0.599	− 0.090
	(1.576)	(0.541)	(1.061)	(0.278)	(0.403)	(0.119)
Month 7	− 9.393***	− 3.106***	− 5.913***	0.530*	− 1.000**	0.096
	(1.780)	(0.609)	(1.143)	(0.298)	(0.494)	(0.111)
Observations	49,140	49,140	49,140	49,140	49,140	49,140
Adjusted R-sq	0.90	0.76	0.90	0.77	0.84	0.46
Mean Dependent	87.12	20.11	53.83	4.87	5.42	2.89
Baseline FE	X	X	X	X	X	X

Source: *Mexico’s National Public Security System* (*Secretariado Ejecutivo del Sistema Nacional de Seguridad Pública*)

Plotted coefficients are event-study dummy variables, *β*_*q*_. Each plotted point represents the number of months before and after the lockdown. Baseline fixed effects are included at the municipality, month, and year. Vertical lines show the start and end of the lockdown. The long-dashed red line shows the month before the start of the lockdown and the short-dashed green line indicates the end of the lockdown. Robust standard errors are clustered at the municipal level. Significance levels: **p* < 0.1, ***p* < 0.05, ****p* < 0.01 Crimes are measured per 100,000 persons

4,

**Table 5:** Event-study specification: low/middle population municipalities (Fig. 3)

	All crime	Assault & battery	Theft & property crime	Fraud	Drug crime & extortion	Homicides
	(1)	(2)	(3)	(4)	(5)	(6)
Month -2	2.513***	0.672	1.400**	0.072	0.182	0.187
	(0.861)	(0.411)	(0.593)	(0.168)	(0.231)	(0.149)
Month 0	− 3.833***	− 0.963**	− 2.354***	− 0.628***	− 0.092	0.204
	(0.909)	(0.426)	(0.603)	(0.163)	(0.179)	(0.157)
Month 1	− 17.274***	− 5.435***	− 9.299***	− 1.520***	− 1.029***	0.009
	(1.094)	(0.482)	(0.709)	(0.190)	(0.217)	(0.156)
Month 2	− 20.109***	− 7.612***	− 10.145***	− 1.483***	− 0.564**	− 0.306**
	(1.244)	(0.544)	(0.828)	(0.175)	(0.223)	(0.149)
Month 3	− 9.381***	− 3.009***	− 4.896***	− 0.934***	− 0.434**	− 0.108
	(1.121)	(0.456)	(0.777)	(0.176)	(0.191)	(0.151)
Month 4	− 8.489***	− 2.273***	− 5.094***	− 0.547***	− 0.571*	− 0.003
	(1.572)	(0.567)	(0.995)	(0.198)	(0.297)	(0.158)
Month 5	− 6.880***	− 2.174***	− 4.038***	− 0.397**	− 0.618**	0.347**
	(1.109)	(0.494)	(0.721)	(0.191)	(0.278)	(0.163)
Month 6	− 5.021***	− 1.593***	− 2.986***	0.147	− 0.517*	− 0.072
	(1.152)	(0.464)	(0.817)	(0.211)	(0.285)	(0.160)
Month 7	− 4.904***	− 1.530***	− 3.020***	0.130	− 0.675***	0.190
	(1.181)	(0.522)	(0.827)	(0.224)	(0.252)	(0.168)
Observations	43,670	43,670	43,670	43,670	43,670	43,670
Adjusted R-sq	0.83	0.67	0.81	0.44	0.70	0.32
Mean Dependent	87.12	20.11	53.83	4.87	5.42	2.89
Baseline FE	X	X	X	X	X	X

Source: *Mexico’s National Public Security System* (*Secretariado Ejecutivo del Sistema Nacional de Seguridad Pública*)

Plotted coefficients are event-study dummy variables, *βq*. Each plotted point represents the number of months before and after the lockdown. Baseline fixed effects are included at the municipality, month, and year. Vertical lines show the start and end of the lockdown. The long-dashed red line shows the month before the start of the lockdown and the short-dashed green line indicates the end of the lockdown. Robust standard errors are clustered at the municipal level. Significance levels: **p* < 0.1, ***p* < 0.05, ****p* < 0.01 Crimes are measured per 100,000 persons

5,

**Table 6:** Event-study specification: high population municipalities (Fig. 3)

	All crime	Assault & battery	Theft & property crime	Fraud	Drug crime & extortion	Homicides
	(1)	(2)	(3)	(4)	(5)	(6)
Month − 2	− 1.444	− 0.632	− 1.378	0.184	0.348	0.034
	(1.153)	(0.429)	(0.842)	(0.203)	(0.263)	(0.114)
Month 0	− 8.121***	− 2.198***	− 5.204***	− 1.113***	0.102	0.293**
	(1.274)	(0.443)	(0.881)	(0.195)	(0.356)	(0.120)
Month 1	− 32.582***	− 9.069***	− 20.273***	− 2.582***	− 0.708	0.051
	(2.068)	(0.575)	(1.326)	(0.308)	(0.539)	(0.123)
Month 2	− 40.009***	− 12.552***	− 23.019***	− 2.895***	− 1.246***	− 0.297**
	(2.277)	(0.649)	(1.603)	(0.303)	(0.376)	(0.118)
Month 3	− 23.737***	− 7.517***	− 13.456***	− 1.948***	− 0.706*	− 0.111
	(1.847)	(0.561)	(1.237)	(0.297)	(0.427)	(0.139)
Month 4	− 18.588***	− 4.923***	− 12.152***	− 0.764**	− 0.785	0.036
	(1.877)	(0.585)	(1.282)	(0.332)	(0.477)	(0.125)
Month 5	− 17.157***	− 5.100***	− 10.842***	− 0.437	− 0.970*	0.193
	(1.732)	(0.549)	(1.238)	(0.314)	(0.555)	(0.124)
Month 6	− 9.996***	− 4.085***	− 5.468***	0.314	− 0.675	− 0.083
	(1.888)	(0.638)	(1.273)	(0.331)	(0.485)	(0.136)
Month 7	− 10.853***	− 3.584***	− 6.836***	0.533	− 1.092*	0.126
	(2.130)	(0.724)	(1.366)	(0.354)	(0.595)	(0.125)
Observations	11,670	11,670	11,670	11,670	11,670	11,670
Adjusted R-sq	0.91	0.81	0.91	0.85	0.86	0.69
Mean Dependent	87.12	20.11	53.83	4.87	5.42	2.89
Baseline FE	X	X	X	X	X	X

Source: *Mexico’s National Public Security System* (*Secretariado Ejecutivo del Sistema Nacional de Seguridad Pública*)

Plotted coefficients are event-study dummy variables, *βq*. Each plotted point represents the number of months before and after the lockdown. Baseline fixed effects are included at the municipality, month, and year. Vertical lines show the start and end of the lockdown. The long-dashed red line shows the month before the start of the lockdown and the short-dashed green line indicates the end of the lockdown. Robust standard errors are clustered at the municipal level. Significance levels: **p* < 0.1, ***p* < 0.05, ****p* < 0.01 Crimes are measured per 100,000 persons

6,

**Table 7:** Event-study specification: low/middle crime municipalities (Fig. 3)

	All crime	Assault & battery	Theft & property crime	Fraud	Drug crime & extortion	Homicides
	(1)	(2)	(3)	(4)	(5)	(6)
Month − 2	− 0.278	− 0.252	− 0.259	0.057	0.002	0.174
	(1.140)	(0.374)	(0.917)	(0.159)	(0.225)	(0.126)
Month 0	− 6.941***	− 2.040***	− 4.192***	− 1.129***	0.072	0.348***
	(1.401)	(0.436)	(1.008)	(0.188)	(0.371)	(0.124)
Month 1	− 26.765***	− 7.458***	− 16.239***	− 2.071***	− 1.121***	0.124
	(1.809)	(0.514)	(1.318)	(0.236)	(0.286)	(0.120)
Month 2	− 32.707***	− 10.687***	− 18.328***	− 2.188***	− 1.292***	− 0.212*
	(1.944)	(0.614)	(1.394)	(0.214)	(0.276)	(0.120)
Month 3	− 19.231***	− 6.270***	− 10.470***	− 1.380***	− 1.001***	− 0.109
	(1.644)	(0.540)	(1.156)	(0.209)	(0.303)	(0.146)
Month 4	− 13.992***	− 3.640***	− 8.732***	− 0.488*	− 1.228***	0.095
	(1.756)	(0.527)	(1.269)	(0.269)	(0.398)	(0.129)
Month 5	− 13.112***	− 4.212***	− 7.811***	− 0.308	− 1.075**	0.294**
	(1.692)	(0.550)	(1.191)	(0.279)	(0.420)	(0.131)
Month 6	− 5.961***	− 3.356***	− 2.395*	0.427	− 0.616	− 0.022
	(1.907)	(0.662)	(1.259)	(0.344)	(0.419)	(0.142)
Month 7	− 6.145***	− 2.682***	− 3.461***	0.550	− 0.715*	0.163
	(2.022)	(0.706)	(1.274)	(0.382)	(0.379)	(0.135)
Observations	21,567	21,567	21,567	21,567	21,567	21,567
Adjusted R-sq	0.90	0.76	0.90	0.70	0.83	0.52
Mean Dependent	87.12	20.11	53.83	4.87	5.42	2.89
Baseline FE	X	X	X	X	X	X

Source: *Mexico’s National Public Security System* (*Secretariado Ejecutivo del Sistema Nacional de Seguridad Pública*)

Plotted coefficients are event-study dummy variables, *βq*. Each plotted point represents the number of months before and after the lockdown. Baseline fixed effects are included at the municipality, month, and year. Vertical lines show the start and end of the lockdown. The long-dashed red line shows the month before the start of the lockdown and the short-dashed green line indicates the end of the lockdown. Robust standard errors are clustered at the municipal level. Significance levels: **p* < 0.1, ***p* < 0.05, ****p* < 0.01 Crimes are measured per 100,000 persons

7,

**Table 8:** Event-study specification: high crime municipalities (Fig. 3)

	All crime	Assault & battery	Theft & property crime	Fraud	Drug crime & extortion	Homicides
	(1)	(2)	(3)	(4)	(5)	(6)
Month − 2	− 1.630	− 1.071*	− 1.369	0.281	0.476	0.053
	(1.650)	(0.556)	(1.248)	(0.262)	(0.336)	(0.138)
Month 0	− 7.440***	− 2.425***	− 4.527***	− 1.225***	0.442	0.296**
	(1.526)	(0.538)	(1.051)	(0.238)	(0.425)	(0.143)
Month 1	− 36.478***	− 9.942***	− 23.065***	− 3.082***	− 0.514	0.125
	(2.526)	(0.701)	(1.583)	(0.404)	(0.672)	(0.143)
Month 2	− 41.829***	− 13.461***	− 23.746***	− 3.269***	− 1.150**	− 0.203
	(2.733)	(0.750)	(1.935)	(0.380)	(0.461)	(0.137)
Month 3	− 24.327***	− 7.856***	− 13.974***	− 1.847***	− 0.594	− 0.056
	(2.138)	(0.645)	(1.455)	(0.355)	(0.494)	(0.155)
Month 4	− 18.812***	− 5.586***	− 12.138***	− 0.537	− 0.589	0.037
	(2.255)	(0.684)	(1.573)	(0.405)	(0.542)	(0.145)
Month 5	− 17.227***	− 5.511***	− 10.986***	− 0.130	− 0.893	0.293**
	(2.032)	(0.637)	(1.477)	(0.378)	(0.658)	(0.139)
Month 6	− 8.926***	− 4.357***	− 4.753***	0.714*	− 0.560	0.030
	(2.340)	(0.780)	(1.570)	(0.402)	(0.578)	(0.149)
Month 7	− 10.139***	− 3.808***	− 6.283***	0.901**	− 1.135	0.186
	(2.646)	(0.873)	(1.697)	(0.418)	(0.729)	(0.135)
Observations	15,629	15,629	15,629	15,629	15,629	15,629
Adjusted R-sq	0.89	0.74	0.88	0.80	0.86	0.61
Mean Dependent	87.12	20.11	53.83	4.87	5.42	2.89
Baseline FE	X	X	X	X	X	X

Source: *Mexico’s National Public Security System* (*Secretariado Ejecutivo del Sistema Nacional de Seguridad Pública*)

Plotted coefficients are event-study dummy variables, *β*_*q*_. Each plotted point represents the number of months before and after the lockdown. Baseline fixed effects are included at the municipality, month, and year. Vertical lines show the start and end of the lockdown. The long-dashed red line shows the month before the start of the lockdown and the short-dashed green line indicates the end of the lockdown. Robust standard errors are clustered at the municipal level. Significance levels: **p* < 0.1, ***p* < 0.05, ****p* < 0.01 Crimes are measured per 100,000 persons

8,

**Table 9:** Event-study specification: States with high relative change in men’s unemployment (Fig. 4)

	All crime	Assault & battery	Theft & property crime	Fraud	Drug crime & extortion	Homicides
	(1)	(2)	(3)	(4)	(5)	(6)
Month − 2	0.722	− 0.090	− 0.095	0.163	0.631	0.113
	(4.304)	(1.575)	(2.640)	(0.360)	(0.569)	(0.168)
Month 0	− 7.646**	− 1.888	− 4.846***	− 1.199***	0.133	0.154
	(2.665)	(1.192)	(1.346)	(0.333)	(0.361)	(0.161)
Month 1	− 32.051***	− 8.993***	− 19.621***	− 3.050***	− 0.410	0.023
	(5.421)	(1.531)	(3.615)	(0.476)	(0.432)	(0.193)
Month 2	− 39.085***	− 12.176***	− 22.431***	− 3.150***	− 1.005**	− 0.322**
	(6.044)	(1.784)	(4.154)	(0.604)	(0.379)	(0.150)
Month 3	− 21.984***	− 6.401***	− 12.862***	− 2.038***	− 0.760	0.077
	(5.064)	(1.457)	(3.266)	(0.373)	(0.454)	(0.150)
Month 4	− 18.286***	− 4.388***	− 11.829***	− 1.255***	− 0.922	0.108
	(4.233)	(1.405)	(2.459)	(0.334)	(0.580)	(0.201)
Month 5	− 15.269***	− 4.347***	− 9.542***	− 0.778*	− 0.788*	0.186
	(3.876)	(1.394)	(2.614)	(0.434)	(0.446)	(0.159)
Month 6	− 7.100*	− 2.314	− 4.491**	0.248	− 0.599	0.055
	(3.444)	(1.454)	(2.061)	(0.549)	(0.506)	(0.189)
Month 7	− 8.179**	− 2.297*	− 5.554**	0.628	− 1.251*	0.295
	(3.438)	(1.268)	(2.279)	(0.641)	(0.687)	(0.170)
Observations	320	320	320	320	320	320
Adjusted R-sq	0.95	0.91	0.94	0.93	0.93	0.95
Mean Dependent	87.12	20.11	53.83	4.87	5.42	2.89
Baseline FE	X	X	X	X	X	X

Source: *Mexico’s National Public Security System* (*Secretariado Ejecutivo del Sistema Nacional de Seguridad Pública*)

Plotted coefficients are event-study dummy variables, *β*_*q*_. Each plotted point represents the number of months before and after the lockdown. Baseline fixed effects are included at the municipality, month, and year. Vertical lines show the start and end of the lockdown. The long-dashed red line shows the month before the start of the lockdown and the short-dashed green line indicates the end of the lockdown. Robust standard errors are clustered at the municipal level. Significance levels: **p* < 0.1, ***p* < 0.05, ****p* < 0.01 Crimes are measured per 100,000 persons

9,

**Table 10:** Event-study specification: States with low relative change in men’s unemployment (Fig. 4)

	All crime	Assault & battery	Theft & property crime	Fraud	Drug crime & extortion	Homicides
	(1)	(2)	(3)	(4)	(5)	(6)
Month − 2	− 2.593	− 0.896*	− 1.696	0.144	− 0.050	− 0.095
	(1.619)	(0.461)	(1.487)	(0.219)	(0.357)	(0.155)
Month 0	− 6.577**	− 1.846***	− 4.259*	− 0.789***	− 0.025	0.343
	(2.936)	(0.548)	(2.233)	(0.231)	(0.508)	(0.198)
Month 1	− 24.913***	− 7.217***	− 15.235***	− 1.518***	− 0.997	0.055
	(4.738)	(1.426)	(2.692)	(0.389)	(0.959)	(0.190)
Month 2	− 30.491***	− 10.195***	− 16.946***	− 1.849***	− 1.245	− 0.255
	(6.882)	(2.262)	(4.351)	(0.284)	(0.723)	(0.199)
Month 3	− 18.685***	− 6.500***	− 10.019***	− 1.287***	− 0.488	− 0.391
	(5.304)	(1.813)	(3.001)	(0.372)	(0.648)	(0.266)
Month 4	− 13.925***	− 4.414***	− 8.832***	− 0.006	− 0.489	− 0.184
	(3.896)	(0.933)	(2.391)	(0.413)	(0.785)	(0.188)
Month 5	− 14.366***	− 4.571***	− 9.120***	0.069	− 0.889	0.144
	(4.450)	(1.324)	(2.381)	(0.519)	(1.014)	(0.183)
Month 6	− 10.184**	− 4.634***	− 5.136*	0.416	− 0.598	− 0.232
	(4.042)	(1.223)	(2.572)	(0.366)	(0.878)	(0.276)
Month 7	− 10.591**	− 3.903***	− 6.269**	0.433	− 0.753	− 0.100
	(4.282)	(1.285)	(2.402)	(0.436)	(1.036)	(0.228)
Observations	320	320	320	320	320	320
Adjusted R-sq	0.95	0.94	0.94	0.88	0.93	0.93
Mean Dependent	87.12	20.11	53.83	4.87	5.42	2.89
Baseline FE	X	X	X	X	X	X

Source: *Mexico’s National Public Security System* (*Secretariado Ejecutivo del Sistema Nacional de Seguridad Pública*)

Plotted coefficients are event-study dummy variables, *β*_*q*_. Each plotted point represents the number of months before and after the lockdown. Baseline fixed effects are included at the municipality, month, and year. Vertical lines show the start and end of the lockdown. The long-dashed red line shows the month before the start of the lockdown and the short-dashed green line indicates the end of the lockdown. Robust standard errors are clustered at the municipal level. Significance levels: **p* < 0.1, ***p* < 0.05, ****p* < 0.01 Crimes are measured per 100,000 persons

10,

**Table 11:** Event-study specification: no Mexico City (Fig. 5)

	All Crime	Assault & Battery	Theft & Property Crime	Fraud	Drug Crime & Extortion	Homicides
	(1)	(2)	(3)	(4)	(5)	(6)
Month − 2	0.061	− 0.290	0.020	0.061	0.263	0.008
	(0.957)	(0.373)	(0.677)	(0.157)	(0.225)	(0.107)
Month 0	− 7.227***	− 1.949***	− 4.659***	− 1.046***	0.132	0.295***
	(1.112)	(0.380)	(0.765)	(0.172)	(0.311)	(0.111)
Month 1	− 26.258***	− 7.744***	− 15.843***	− 2.047***	− 0.658	0.034
	(1.764)	(0.508)	(1.149)	(0.227)	(0.469)	(0.114)
Month 2	− 32.237***	− 10.670***	− 17.911***	− 2.208***	− 1.171***	− 0.276**
	(1.903)	(0.576)	(1.357)	(0.212)	(0.327)	(0.110)
Month 3	− 18.509***	− 6.140***	− 10.216***	− 1.503***	− 0.473	− 0.177
	(1.577)	(0.510)	(1.054)	(0.196)	(0.366)	(0.127)
Month 4	− 15.482***	− 4.365***	− 10.041***	− 0.522**	− 0.524	− 0.029
	(1.678)	(0.527)	(1.146)	(0.255)	(0.394)	(0.115)
Month 5	− 14.203***	− 4.408***	− 9.091***	− 0.207	− 0.676	0.180
	(1.558)	(0.490)	(1.092)	(0.261)	(0.472)	(0.116)
Month 6	− 8.029***	− 3.154***	− 4.688***	0.284	− 0.379	− 0.091
	(1.654)	(0.566)	(1.093)	(0.275)	(0.412)	(0.125)
Month 7	− 9.555***	− 3.028***	− 6.241***	0.404	− 0.776	0.085
	(1.881)	(0.644)	(1.179)	(0.307)	(0.507)	(0.115)
Observations	48,820	48,820	48,820	48,820	48,820	48,820
Adjusted R-sq	0.90	0.76	0.90	0.60	0.85	0.46
Mean Dependent	87.12	20.11	53.83	4.87	5.42	2.89
Baseline FE	X	X	X	X	X	X

Source: *Mexico’s National Public Security System* (*Secretariado Ejecutivo del Sistema Nacional de Seguridad Pública*)

Plotted coefficients are event-study dummy variables, *β*_*q*_. Each plotted point represents the number of months before and after the lockdown. Baseline fixed effects are included at the municipality, month, and year. Vertical lines show the start and end of the lockdown. The long-dashed red line shows the month before the start of the lockdown and the short-dashed green line indicates the end of the lockdown. Robust standard errors are clustered at the municipal level. Significance levels: **p* < 0.1, ***p* < 0.05, ****p* < 0.01 Crimes are measured per 100,000 persons

11.

## Footnotes

1 There is suggestive evidence that some criminal organizations diversify their portfolio by engaging in kidnapping (Bergman, 2018) and extortion (Magaloni et al., 2011).

2 Oster applies this methodology to a sample of papers published in the *American Economic Review*, *Quarterly Journal of Economics*, *The Journal of Political Economy*, and *Econometrica* from 2008–2010. She found that using this bounding methodology allowed 90% of the randomized and 50% of the nonrandomized results to continue being statistically significant.
